# Obituary

**Published:** 1912-03-30

**Authors:** 


### Obituary.

Dr. Draper Mackinder, M.D., a retired practitioner of Hove, has died at the age of ninety-three. He became qualified in 1847 at the Royal College of Surgeons, after pursuing his medical studies at St. Bartholomew's Hospital and at Glasgow. In 1853 he became M.D. of St. Andrews, and four years later a Fellow of the Royal College of Surgeons of Edinburgh.

Dr. Allen Abraham Duke, of Southwick, Sussex, has died suddenly. He was formerly one of the medical officers attached to H.M. Convict Prison at Portsmouth. He qualified at Edinburgh in 1858, obtaining the M.D. of that University in 1859. He spent some time studying in the Paris schools. Dr. Duke held the appointment of surgeon to the North Surrey Schools at Anerley.

Dr. Eugene Stephen Yonge, of Manchester, a well-known authority on the diseases of the nose and throat, has died after a lingering illness. Dr. Yonge graduated at Edinburgh no longer ago than 1891, taking the M.D. in 1897. He studied also for a considerable time on the Continent, especially in Vienna and Paris. Only last year he attended the International Hygiene Exhibition at Dresden as the English representative in the Hay Fever section. Dr. Yonge was a practical advocate of the method of treating asthma and hay fever by resection of the nasal nerve. His handbook on "Diseases of the Nose and Throat" is a recent and valuable addition to the text-books on this specialty. Among his earlier appointments were those as resident at the South Devon Hospital, Plymouth, and the Southern Hospital for Women, Manchester. Dr. Yonge was physician to the Crossley Sanatorium and the Manchester Hospital for Consumption and Diseases of the Throat.

Dr. James Snowden-Smith, of Tavistock, Devon, has died very suddenly while away on a visit. He had a very large practice in Tavistock, and held a number of public appointments in the district, including that of honorary surgeon to the local cottage hospital. Dr. Snowden-Smith was fifty-seven years of age, and had been in practice in Tavistock for over twenty years. Dr. Snowden-Smith was a student at Guy's Hospital, and qualified as member of the Royal College of Surgeons, London, in 1880, being afterwards admitted L.R.C.P. of Edinburgh. Before settling in Tavistock he held a resident appointment at the General Infirmary at Salisbury.

Dr. M. Ogilvy-Ramsay, of Carlisle, honorary surgeon to the Cumberland Infirmary, has died at the age of 48. He was born in the village of Kirriemuir, immortalised by Barrie as "Thrums," and at first joined the University of St. Andrews. He took his medical degrees at Edinburgh, including the Fellowship of the Royal College of Surgeons. Among the hospital appointments held by Dr. Ogilvy-Ramsay were those as house surgeon at the Royal Infirmary, Edinburgh, and at the Children's Hospital, Liverpool. He then settled in Carlisle, and devoted special attention to obstetric medicine. Ten years later he was elected on the surgical staff of the Cumberland Infirmary. An effort will be made to commemorate by a permanent record the splendid work done by Dr. Ramsay in the town of Carlisle.

